# Anti-Mullerian-Hormone during pregnancy and peripartum using the new Beckman Coulter AMH Gen II Assay

**DOI:** 10.1186/s12958-015-0082-4

**Published:** 2015-08-07

**Authors:** A. Köninger, B. Schmidt, P. Mach, D. Damaske, S. Nießen, R. Kimmig, T. Strowitzki, A. Gellhaus

**Affiliations:** Department of Gynecology and Obstetrics, University of Duisburg-Essen, Hufelandstrasse 55, 45122 Essen, Germany; Institute for Medical Informatics, Biometry and Epidemiology (IMIBE), University of Duisburg-Essen, Hufelandstrasse 55, 45122 Essen, Germany; Department of Gynecological Endocrinology and Reproductive Medicine, University of Heidelberg, Voßstrasse 9, 69115 Heidelberg, Germany; Department of Gynecology and Obstetrics, University of Heidelberg, Voßstrasse 9, 69115 Heidelberg, Germany

**Keywords:** Anti-Mullerian-Hormone, pregnancy, peripartal, conventional Beckman Coulter AMH Gen II assay, modified Beckman Coulter AMH Gen II assay

## Abstract

**Background:**

AMH levels determined by the conventional AMH assay declined during pregnancy and postpartum. A new Beckman Coulter AMH Gen II assay removes the potentially assay-interfering complement which is activated in pregnancy. The aim of this study was to evaluate if the decline of AMH levels in the serum of pregnant women during the course of pregnancy and peripartum was assay-dependent and thus artificial.

**Methods:**

In this cross-sectional study prepartal blood samples were collected from 62 patients (median age 30.6 years [interquartile range: 25.6 - 34.5]) in the third trimester of pregnancy and again 1–4 days after delivery between 2011 and 2012. In another cohort of 11 patients (median age 34.1 years [interquartile range: 32.6 - 37.8]) blood samples were taken in different trimesters of pregnancy between 1995 and 2001. The conventional and the modified AMH assay were performed in the same patient serum samples. We used the conventional and the modified AMH-Gen-II ELISA (Beckman Coulter, Immunotech, Webster, USA) for the assessment of AMH levels. The Wilcoxon signed rank test was used for determining differences between AMH levels pre- and postpartum. The method of Bland and Altman was applied for analyzing the agreement of both methods for determining AMH levels.

**Results:**

AMH values peripartum were lower than those expected in fertile non-pregnant women of comparable age. An overall mean difference of 0.44 ng/ml was observed between the conventional and the modified assay. Measurements with the modified assay showed a significant decline of postpartal levels compared with prepartal levels which is consistent with values obtained using the conventional assay (both *p* < 0.00001). Compared to the longitudinal measurements of AMH levels determined using the conventional assay, AMH levels obtained using the modified assay suggest a steeper decline of values during the course of pregnancy.

**Conclusion:**

By comparing the conventional assay for AMH determination with the modified assay the present study confirmed that AMH levels decline during the course of pregnancy and early after delivery.

## Background

Anti-Mullerian Hormone (AMH) is known to be the most precise predictor of ovarian reserve in women [1, 2]. It is produced by the granulosa cells of small antral and preantral follicles and reflects the size of the pool of these follicles [3, 4]. AMH declines with age [5] but remains stable during the menstrual cycle [6, 7] allowing its determination at random [8]. We have recently observed that AMH decreases during pregnancy, showing the lowest values peripartum, followed by a significant increase of AMH during the first four days after delivery [9]. We concluded that the extremely hypogonadotropic status in pregnancy could be responsible for the decline of AMH during pregnancy, followed by a rapid recovery after delivery. In the respective study, we used the Beckman Coulter Gen II Assay which was available until July 2013. Recent studies have suggested that storage conditions led to fluctuations of AMH values determined by this assay, whereas a modified AMH Beckman Coulter Gen II Assay (available since July 2013) resulted in more stable values of AMH independent of storage time and conditions [10, 11]. Additionally, the AMH levels determined by the modified assay are reported to be higher [11]. According to the manufacturer, this effect may reflect a possible interaction of complement with the conventional assay resulting in fluctuations due to individual complement activation during storage. The modified assay includes a pre-mixing step with a highly anionic buffer which removes this complement.

It is thought that pregnancy is associated with complement activation [12–15] that may lead to a more pronounced difference of AMH determined by the conventional assay compared to the modified assay in pregnant women as a result of sample handling and storing conditions [11]. According to these observations, decreased AMH levels peripartally may simply be an artificial phenomenon.

In the present study we have determined AMH levels during pregnancy and peripartum with the new modified AMH assay in a subgroup of our study population in which the conventional assay was recently used to determine AMH values [9]. The aim was to explore whether the low prepartal levels of AMH may have been assay-dependent and artificial due to progressive complement activation in the course of pregnancy.

## Methods

### Study population

Prepartal blood samples were collected from 62 patients aged between 18 to 41 years (median age 30.6 years [interquartile range (IQR): 25.6 - 34.5]) in the third trimester of pregnancy as the patients presented for labour and again 1–4 days after delivery. None of the patients included in this study exhibited a history of infertility, operations on the ovaries, chemotherapy or radiation in the past. All women were examined between 2011 and 2012. Informed written consent was obtained from all women and the study was approved by the local research ethics committee (number 11–4643).

Blood samples were taken between October 10^th^, 2011 and February 21^th^, 2012. The conventional assay was used in frozen samples between November 9^th^, 2011 and February 27^th^, 2012. The modified assay was performed using frozen aliquots of the same patient samples on July 18^th^, 2014.

In another cohort of 11 patients aged between 29 to 39 years (median age 34.1 years [IQR: 32.6 - 37.8]) who presented in our clinic between 1995 and 2001, we took blood samples during various trimesters of pregnancy and evaluated AMH levels at several points during the course of the pregnancy.

The determination of values with the conventional assay took place between December 6^th^, 2011 and February 12^th^, 2012. The determination of values with the modified assay took place on August 8^th^, 2014.

All patients included in the present study were part of the study populations used for our recently published study [9]. Aliquots from identical blood samples were used for both studies. As no additional aliquots were available for some participants of the original study population, only 62 out of the original 69 and 11 out of the original 15 patients were included in the present study. No substantial differences in AMH levels (assessed using the conventional assay) were detected between the original study population and the subgroup included in the present study.

### Sampling of blood serum

Blood samples (9 ml) were collected from each woman using S-Monovettes (Sarstedt AG & Co.), immediately stored at 4 °C and processed within 4 h to avoid blood cell lysis. Blood fractionation was carried out by centrifugation for 10 min at 2500xg. Subsequently, 3 to 4 ml of the upper phase constituting blood serum were removed for the assessment of AMH levels. Samples were pipetted into 4 aliquots and stored at −80 °C.

### Determination of AMH levels with the conventional assay

As previously stated [9], we first used the enzymatically amplified two-site AMH-Gen-II ELISA (Beckman Coulter, Immunotech, Webster, Texas, USA) which was available until July 2013. Undiluted serum samples and controls were dispensed into the wells which were coated with anti-AMH antibody, followed by the addition of the anti-AMH detection antibody labelled with biotin. 100 μl of the streptavidin-horseradish peroxidase (HRP) was added after washing, followed by the addition of 100 μl of substrate solution containing TMB for 8–12 min. Using an automatic ELISA reader (Bio-Rad, Hercules, CA) the degree of enzymatic turnover of the substrate was determined by dual wavelength absorbance measurement at 450 nm and between 600 and 630 nm. The absorbance measured was directly proportional to the AMH concentration in the samples which was calculated from the calibration curve. The results were expressed in ng/ml. Concentrations below 0.08 ng/ml were considered undetectable.

### Determination of AMH levels with the modified assay

The revised test procedure includes an additional assay step before adding AMH Gen II Calibrators, controls, or AMH samples to the microplate. This additional step eliminates the complement interference.

Before adding a sample to the AMH Gen II ELISA microplate, all calibrators, controls, and samples were prepared with the AMH Gen II Assay Buffer (A56021) as follows. In a sample tube, 1 part of each calibrator, control, or test sample respectively was thoroughly mixed with 5 parts AMH Gen II Assay Buffer (for example, 60 μL calibrator, control, or sample + 300 μL AMH Gen II Assay Buffer). No dilution factor was required. Within one hour, 120 μL of the premixed calibrators, controls and samples were added to the appropriate wells and the test proceeded like the conventional AMH Gen II assay.

### Statistical analysis

AMH levels were found not to be normally distributed in the study populations (based on visual inspection and Shapiro-Wilks test; p < 0.0001). Thus, results are reported as median and interquartile ranges. Differences between AMH levels pre- and postpartum were analyzed using the Wilcoxon signed rank test with the level of statistical significance set at α = 0.05. Agreement of methods for determining AMH levels was analyzed using the method of Bland and Altman [16]. Statistical analyses and boxplots were performed using the R statistical package version 3.0.2 [17]. All other plots were created by using SPSS version 20 [18].

## Results

### AMH levels pre- and postpartum

**Fig. 1:**
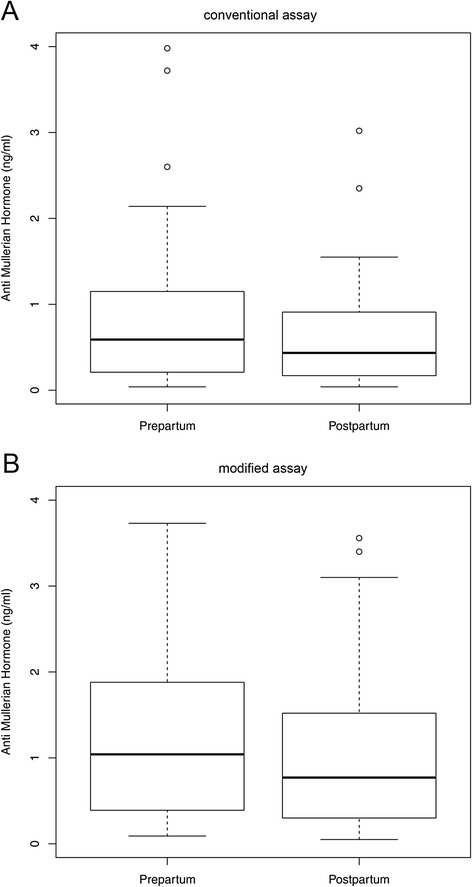
**a**: Box plots illustrating the distribution of AMH levels in women with AMH measurements prepartum and postpartum assessed using the enzymatically amplified two-site AMH-Gen-II ELISA (*n* = 62). Wilcoxon signed rank test: *p* < 0.00001. **b**: Box plots illustrating the distribution of AMH levels in women with AMH measurements prepartum and postpartum assessed using the Beckman Coulter Gen II assay (*n* = 62). Wilcoxon signed rank test: *p* < 0.00001

Compared to the conventional assay, measurement of AMH values with the modified assay resulted in higher values with a median level of 1.04 ng/ml (IQR: 0.40 – 1.87) prepartal and 0.77 ng/ml (IQR: 0.31 – 1.52) postpartal (Table 1). Measurements with the modified assay showed a significant decline of postpartal levels compared to prepartal levels which is consistent with values obtained using the conventional assay (both *p* < 0.00001) (Fig. 1, Table 1).

**Table 1:** AMH values determined with the conventional and the modified assay (Median and IQR)

*N* = 62	AMH prepartal in ng/ml	AMH postpartal in ng/ml	*p*-value
conventional assay	0.59 (0.22 – 1.13)	0.44 (0.18 – 0.91)	<0.00001
modified assay	1.04 (0.40 – 1.87)	0.77 (0.31 – 1.52)	<0.00001

### Agreement of methods

**Fig. 2:**
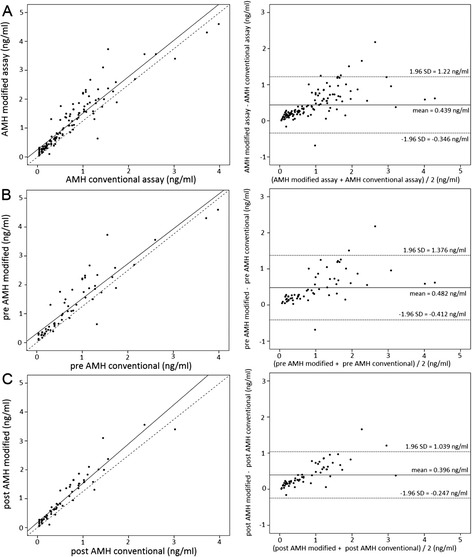
**a:** Bland–Altman plots (incl. mean differences and 95 % limits of agreement) to illustrate differences in AMH between the conventional and the modified assay (*N* = 62). Scatter plots including linear regression lines and lines of equality illustrate the difference between values determined with both assays pre- and postpartum. **b:** Bland–Altman plots (incl. mean differences and 95 % limits of agreement) to illustrate differences in prepartal AMH levels between the conventional and the modified assay (*N* = 62). Scatter plots including linear regression lines and lines of equality illustrate the prepartum difference between values determined with both assays. **c:** Bland–Altman plots (incl. mean differences and 95 % limits of agreement) to illustrate differences in postpartal AMH levels between the conventional and the modified assay (*N* = 62). Scatter plots including linear regression lines and lines of equality illustrate the postpartum difference between values determined with both assays

Figure 2 shows scatter plots including linear regression lines and lines of equality as well as the results of the Bland-Altman analyses including mean differences and 95 % limits of agreement between both methods. An overall mean difference of 0.44 ng/ml is observed between the conventional and the modified assay (Fig. 2a) when pre- and postpartal AMH measurements are combined. The results suggest that differences between both assays increase with rising AMH values irrespective of whether AMH was assessed pre- or postpartally. This is also reflected by the lower mean difference and the smaller limits of agreement observed for postpartal AMH levels compared to prepartal, as postpartal AMH levels were on average lower than those measured prepartal (Fig. 2b and c).

### AMH levels during the course of pregnancy

**Fig. 3:**
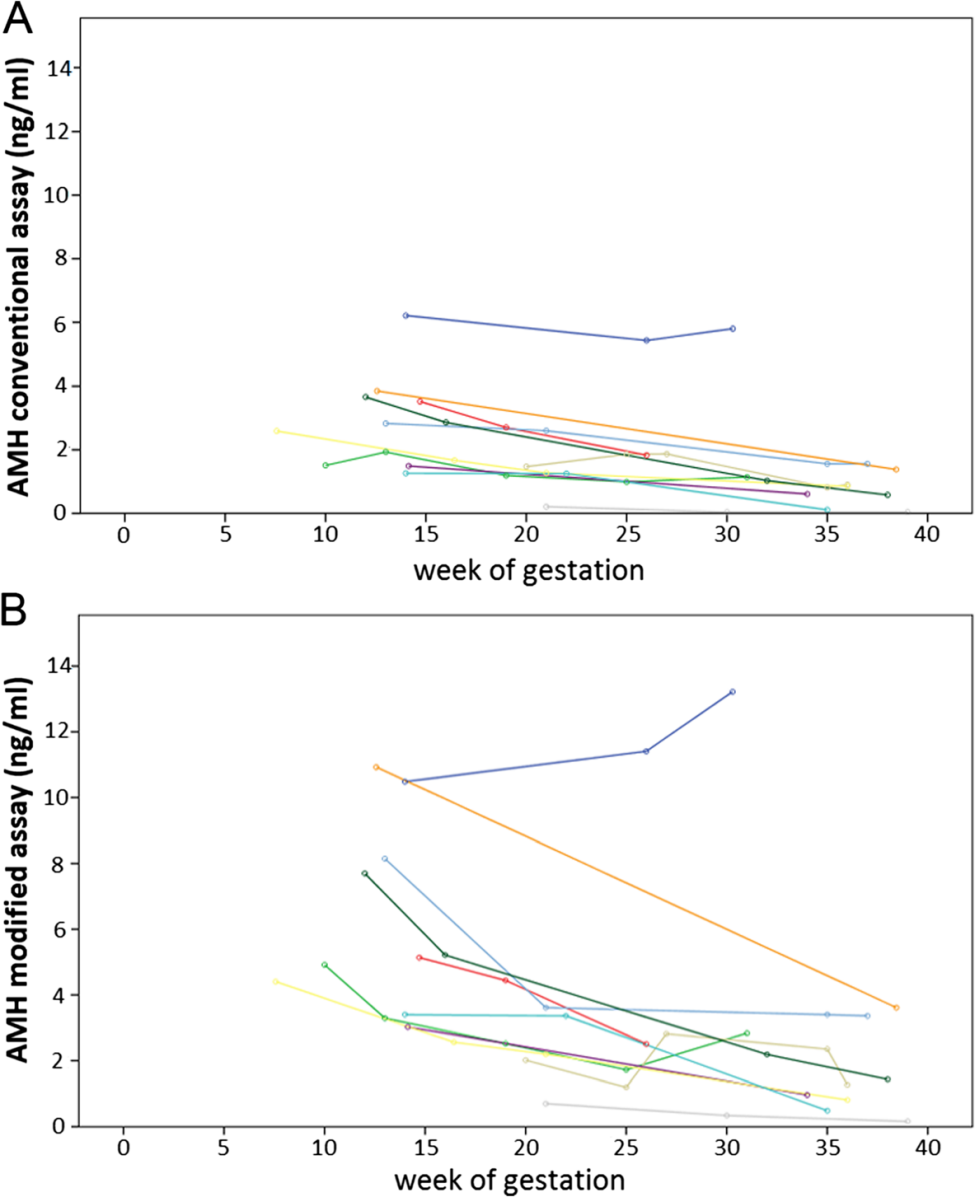
**a:** Longitudinal measurement of AMH levels determined with the conventional assay. **b:** Longitudinal measurement of AMH levels determined with the modified assay

Compared to the longitudinal measurements of AMH levels determined using the conventional assay (Fig. 3a) graphical analysis of AMH levels determined using the modified assay suggests a steeper decline of values during the course of pregnancy (Fig. 3b).

## Discussion

The aim of this study was to investigate whether decreasing prepartal levels of AMH reported previously may have been assay-dependent and thus artificial. As recently reported by us [9] AMH levels declined with ongoing gestational age until the first day postpartum. This decline was questioned as being artificial, since to the development of a modified assay, available since July 2013, eliminated the complement and resulted in less storage-dependent AMH fluctuations as well as consistently higher AMH values. It was supposed that a serological factor like complement was able to interfere with the conventional assay and individual complement activation resulted in fluctuations, whereas no consistent fluctuation patterns were described with the modified assay [11]. According to the manufacturer’s instructions, the consistently higher values determined with the modified assay were attributable to a step during the assay procedure whereby the effects of complement were eliminated. Since complement is activated in pregnancy, the decline described in our recently published study could be considered as artificial due to the complement binding action of the assay.

As shown by us, AMH values determined using the conventional as well as the modified assay seem to decline in the course of pregnancy and peripartum. Peripartal AMH values were lower than those expected in fertile non-pregnant women of comparable age [5]. The determination at the end of the third trimester resulted in very low values with both assays, reflecting the gestational age associated decline. The ongoing decline early after delivery was confirmed by the modified assay. The overall difference between the values remained higher with the modified assay for both pre- and postpartal determinations. Furthermore, our results indicate that the differences between the conventional and modified assay are not independent of the magnitude of the AMH values in advanced stages of pregnancy as well as during the first four days postpartum. Thus, the decline in AMH levels during pregnancy is expected to be more pronounced using the modified assay, as high AMH levels in the early stages of pregnancy would be more affected by the induced AMH increase.

We have hypothesized [5] that the hypogonadotropic status may be responsible for the AMH decline. Recently published data demonstrated that in hypogonadotropic amenorrhoea AMH levels were not decreased [19]. Another study has examined the relationship between AMH, gonadotropins, estradiol and progesterone in the first and second trimester and did not found associations [20]. Therefore, it may be possible that additional factors (e.g., the FSH-antagonist follistatin or products of the placenta which are increasing during pregnancy [21]) are contributing to the pregnancy-associated and reversible AMH-decline.

Recently published studies showed meaningful AMH fluctuations depending on storage time at room temperature with an increase in values after 8 h [11]. The management of our processing procedure of samples was very strict with a storage time at room temperature of less than 4 h and storage at −80 °C immediately after processing. None of the samples was frozen twice or more. Therefore, the strict management of sample handling and storage conditions may have contributed to the consistent results of the two assays. The storage times at −80 °C for samples of the longitudinal measured patients varied between 10 and nearly 20 years before AMH was determined using the conventional assay in 2011/2012 and using the modified assay in 2014, respectively. This has to be mentioned together with the small sample size of 11 patients as limitations of our longitudinal study. Storage-dependent fluctuations of AMH have not been investigated so far for storage-times of more than 30 weeks [11]. Thus, we cannot rule out that storage time and conditions had an impact on our results. Our study design was not suitable to clarify the storage-dependent variance, however, we did not observe any indication of storage-dependent differences in the tendency of AMH declining during pregnancy.

In contrast, the tendency of AMH levels determined with the conventional and the modified assay was comparable with the decline in pregnancy as well as postpartum.

In addition, a dilution step to eliminate the complement (AMH Gen II Assay Buffer (A56021)) is part of the modified assay but not of the conventional assay method. To ensure comparability of our results to those of further studies we strictly followed the manufacturer’s instructions in performing both assays which included methods of the respective dilution procedure. However, investigating the exact differences between the applied methods was not the aim of the study, but rather to show that the decline of AMH levels in pregnancy as well as postpartum was present irrespective of the assays applied for AMH determination and the preanalytical step for complement removal. However, new commercially available assays may be able to further improve AMH assessment in the future.

## Conclusion

Since complement is activated in pregnancy, an artificial decline of AMH during the course of pregnancy could not be ruled out when determination was done with the conventional assay as recently published by us.

This study using the modified new Beckman Coulter AMH Gen II Assay which removed the potentially assay-interfering complement verified the AMH decline during the course of pregnancy and early after delivery. According to our data, the AMH decline in pregnancy was not artificial.
